# Study on Hydrogen Diffusion Behavior during Welding of Heavy Plate

**DOI:** 10.3390/ma13173887

**Published:** 2020-09-03

**Authors:** Jianguo Yang, Guohao Liu, Wenjian Zheng

**Affiliations:** College of Mechanical Engineering, Zhejiang University of Technology, Hangzhou 310014, China; yangjg@zjut.edu.cn (J.Y.); 17857685277@163.com (G.L.)

**Keywords:** hydrogen diffusion, welding, heavy plate, numerical simulation, residual stress

## Abstract

For the multi-layer and multi-pass welding process of the heavy plate, the hydrogen diffusion behavior was numerically simulated to study the effect of solid-state phase transition (SSPT) on the hydrogen diffusion in the thickness direction, and the influence of the residual stress-induced diffusion after SSPT. The calculation results were compared with the experimental results. The comparison shows that the distribution of hydrogen concentration in the direction of thickness was in good agreement. The position with the most severe cold cracking sensitivity was located at a 20–30 mm depth from the top surface in this article. After welding, the hydrogen concentration in this position was kept at a high level for a long time under the effect of the size-constraint effect of the heavy plate and the existence of welding residual stress gradient. In addition, the SSPT reduced the residual stress level of weld metal (WM) significantly, increased that of the heat affected zone (HAZ), and the hydrogen was redistributed under the influence of stress. In the process of phase transformation, the parameters of hydrogen diffusion property of the material changed dramatically in a short time, the hydrogen diffusion coefficient increased in order of magnitude, and the solubility decreased in order of magnitude. This directly led to the upward diffusion of hydrogen in WM, and produced a self-gathering effect. For a welded joint of heavy plate, the self-gathering effect between passes was effective in the short-range and ineffective in the long-range.

## 1. Introduction

The hydrogen-assisted cold cracking (HACC) is one of the most common phenomenon in welded joints of high strength steel (HSS). Because of its delayed cracking characteristics, it will pose a major threat to structural safety. HACC of welding is mainly caused by the union effect of three factors in welded joints, i.e., hardened microstructures, diffusion hydrogen enrichment, and high residual stress state. During the formation of welded joint, especially for HSS, the three factors change with time in a complex and variable way, and influence each other. At present, the investigations on HACC mainly focus on the three factors. In addition, there was no effective in-situ test method to detect the hydrogen concentration in the welded joint. Many studies have focused on the effect of hydrogen on welded joint after welding rather than during welding. That mastering the formation mechanism of HACC is the fundamental task of controlling the cold cracking in welded joints. It is necessary to study hydrogen diffusion behavior in the micro or even smaller scale. This direction has always been concerned by the industry around the world, and many research results [1] have been achieved.

The earliest theory of hydrogen enhanced decohesion (HEDE) was first proposed in 1926 by Pfeil [2], who proposed that hydrogen decreased cohesion across cubic cleavage planes and grain boundaries. Later, HEDE theory was improved by researches [3,4,5,6] which suggested that the weakening of the interatomic bond was due to hydrogen 1 s electrons being supplied to the unfilled 3d shell of the iron atom and the decohesion hypothesis–including charge-transfer and weakening of interatomic bonds. So, the tensile separation of atoms (decohesion) was subsequently quantitatively developed in comparison to slip preferentially. Later, hydrogen enhanced localized plasticity (HELP) [7] held that hydrogen has a shielding effect on the elastic interaction between dislocations and obstacles, which leads to the reduction of their hindrance energy, so dislocations become easier to move. In addition, relevant scholars have recently made contributions to the research on hydrogen embrittlement of steel [8,9], mainly studying the hydrogen embrittlement behavior of super duplex steel, low carbon structural steel, and the property changes caused by hydrogen embrittlement behavior.

According to Beachem [10], the micro deformation at the crack tip was affected by the enrichment of hydrogen in the lattice position at the front of the crack tip. Reducing the stress intensity factor would lead to the gradual decrease of micro-plastic deformation. With the change of stress intensity factor and hydrogen concentration, that cracking and fracture morphology of hydrogen-induced cracking showed different characteristics. In addition, there were two kinds of forces of constraint on the welded joint. One was the external binding force caused by the external loads or rigid restraints on the welded joint, and the other was the welding residual stress caused by the non-uniform heat input. The hydrogen diffusion can be induced by stress, so the forces of constraint in the welded joint should be reduced as much as possible. Deng [11] studied the effect of solid-state phase transition (SSPT) on welding residual stress in detail. Considering the martensitic phase transformation of low carbon steel and medium carbon steel in the process of cooling after welding, the residual stresses of the welded joint about two kinds of steel were calculated respectively by establishing the relationship between the phase change expansion and phase variables with thermal cycle curve of the welding. The results showed that the SSPT in low carbon steel has little effect on the welding residual stresses, while the SSPT in high carbon steel obviously changes the residual stress distribution of the welded joint. Phase transformation expansion results in significant compressive stress in the center of the weld.

Wang et al. obtained the proportions of various phase components of the welded joint and predicted the distribution of welding residual stress of ferritic HSS by means of finite element calculations [12,13]. The calculated results were in good agreement with the experimental results. The results showed that more accurate residual stress results can be obtained when isotropic dynamic hardening constitutive relation was adopted and the effect of SSPT was considered. The results of multi-pass welding also verified the conclusion. In addition, the effects of microstructure, stress state and defects on hydrogen diffusion are necessary to study HACC. The diffusion behavior of hydrogen in steel was studied and it was found that by changing the ratio of grain boundary to grain size, the results showed that the grain boundary has a double effect on the hydrogen diffusion coefficient, that was, too small or too large grain size will reduce the hydrogen diffusion rate. Hydrogen diffusion was affected by the grain size, rolling state, and elongated orientation of the grains. At the micro scale, the crystal orientation of each grain also affects the hydrogen diffusion behavior [14,15]. Ilin et al. [16] studied the effect of microstructure induced stress-strain heterogeneity on the hydrogen distribution considering the anisotropy of grains. The polycrystalline microstructure of 316 L stainless steel was synthesized and placed in a hydrogen environment. The distribution of hydrogen was studied by applying mechanical loading. It was found that the stress gradient caused by the anisotropy of polycrystals at the mesoscale result in the redistribution of hydrogen, and the strain rate determined the redistribution of hydrogen. Świerczyńska et al. [17] reported the effect of underwater wet welding parameters and conditions on the diffusible hydrogen content in deposited metal for welding with a self-shielded flux cored wire. The diffusible hydrogen content in deposited metal was determined using the glycerin method according to the Plackett–Burman design, determining the significance of the effect of the stick out length, welding current, arc voltage, travel speed, and water salinity.

Wang et al. [18] reported the effect of quenching-tempering (QT) treatment on the hydrogen embrittlement (HE) resistance of a Cr-Mo-V high strength steel was studied, and tests showed that HE sensitivity decreased to a negligible level after QT treatment. The improvement of HE resistance was mainly attributed to the decreased number of M3C carbides which act as the reversible trapping sites for hydrogen. Rhode et al. [19] studied Cr-Mo-V steel T24 (7CrMoVTiB10-10) base material and TIG weld metal were investigated. The results showed two different effects. At first, the microstructure effect on trapping was evident in terms of higher hydrogen concentrations in the weld metal and increased activation energy for hydrogen release. Secondly, the trap character in case of the base material changed to irreversible at decreased temperature. Abe used experimental and numerical methods to measure hydrogen diffusivity and the influence of DHT on Cr-Mo-V steel welds, they demonstrated that even at temperatures as low as 280 degrees C held over longer time periods, and there was an equivalent dehydrogenation effect as in the existing conditions [20].

In conclusion, Koyama et al. [21] gave an overview of recent progress in microstructure-specific hydrogen mapping techniques. However, the hydrogen diffusion inside the welded joint cannot be in-situ measured by experimental methods including traditional test methods [22,23,24,25] and advanced in-situ observation technology [26,27,28]. Finite element analysis was an effective method to quantitatively predict the change of hydrogen concentration of the welded joint. To date, there is no report about the precise effect of SSPT on hydrogen diffusion in welding. Because of the importance of hydrogen diffusion, we should carefully study the influence mechanism of SSPT effect on hydrogen diffusion and its subsequent influence. The study of hydrogen diffusion behavior and the prevention and control of cold cracking are of great significance for industrial safety. In this study, aiming at the multi-pass welding process of heavy plate, the hydrogen diffusion behavior in the welding process was simulated to study the effect of SSPT on the hydrogen diffusion in the direction of plate thickness, and what did hydrogen diffuse and accumulate under the existence of welding residual stress gradient in the heavy plate.

In engineering, the field with large welding workload were concentrated in shipbuilding industry, nuclear industry and pressure vessels. The welded joint of heavy plate needs to meet the requirements of large load-bearing, high structural stability, and the characteristics of large cost and long cycle in the manufacturing process, so the requirements for the welding quality of welded joints are very high. One of the most important things is to prevent the cold cracking in welding. In order to avoid the cold cracking, we should adopt a very strict welding procedure to reduce the residual hydrogen content in the welding joint. Therefore, the welding of heavy plate was focused in this work to explore the hydrogen diffusion mechanism in the welding process and provide theoretical basis for engineering problems.

## 2. Materials and Experiment

**Figure 1:**
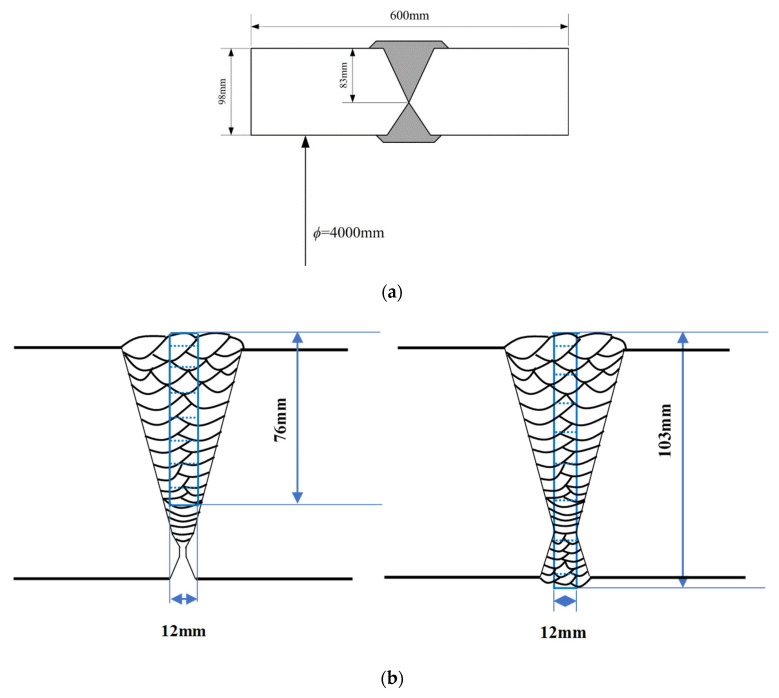
Welded joint size and detection position of residual hydrogen concentration of heavy plate welded joint: (**a**) Welded joint size; (**b**) detection position of residual hydrogen concentration [20].

Segmented butt welded joint for heavy plate of hydrogenation reactors as object of research. The dimensions of the weldment and welded joint are shown in Figure 1a. The length of the heavy plates is 600 mm, the thickness is 98 mm, and the distance between the junction of positive and negative bead and the upper surface is 83 mm. ∅=4000 mm refers to the inner diameter of the hydrogenation reactor made from the heavy plate.

The base metal (BM) was 2.25Cr-1Mo-0.25V steel. The steel of 2.25Cr-1Mo-0.25V is a new material to meet the requirements of oversize hydrogenation reactor and coal liquefaction engineering. Compared with the traditional Cr-Mo steel, its strength is better, the service temperature is higher, the weight reduction is significant, the cost is lower.

The welding method was submerged arc welding (SAW). The welding process parameters were welding current 580 A, arc voltage 32 V, welding speed 60 cm/min, the heat efficiency 0.8–0.9 [20], preheating temperature 200 °C before welding, and inter-pass temperature not more than 250 °C.

The test process of welded joint and the results of hydrogen content detection in welded joint refer to [20]. In the hydrogen measurement test, the double V-shaped groove of 2.25Cr-1Mo-0.25V steel (SA542D Type D Class 4a) was used to welding by SAW process at practical welding condition. After this welding, one of the test coupons (V test coupon) was quenched with ice water to reduce the temperature from 200 °C and then immersed in dry ice–ethanol solution immediately. The welded joint temperature was always kept below −70 °C in order to prevent hydrogen fuse. As for the X test coupon, Dehydrogenation heat treatment (DHT) was performed under the condition of 280 °C × 6 h after one side of v-groove was welded. After that, a back gouging was performed, followed by a magnetic particle test, which confirmed the absence of defects. The test coupon was preheated at 200 °C and above, welded on the gouged side, and then quenched and maintained at below −70 °C.

Next, the two test coupons were processed as follows: As-welded, undergoing 350 °C × 4 h of DHT and undergoing 280 °C × 6 h of DHT, respectively. Wire cutting was carried out according to the position in Figure 1b, that was specimens for measuring hydrogen concentration were collected through the thickness direction at the centre portion of the weld metal. After cutting into several sections, the average hydrogen content of each section was measured by the gas chromatograph method.

## 3. Numerical Modelling

### 3.1. Numerical Approach

**Figure 2:**
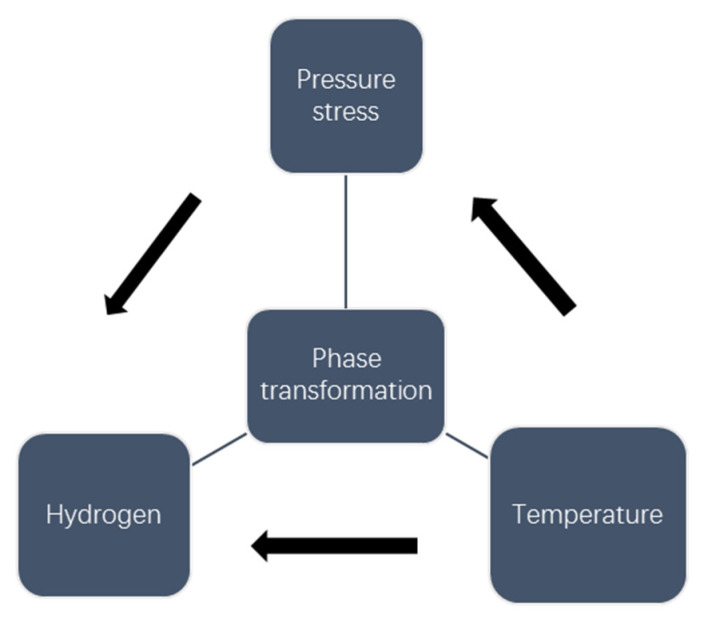
Simulation calculation process of multi-pass welding.

The coupling model of thermal-mechanical-hydrogen diffusion was established for the multi-pass welding process of heavy plate, the schematic process of calculation is shown in Figure 2. Firstly, the thermal process of welding was calculated, and the radiation and convection on the outer surface were used to account for the heat loss in welding. Secondly, the evolution of stress and strain was calculated by using Von Mises yield criterion and elastic-plastic mechanics analysis of related hardening rules. The temperature history obtained in the previous step was added to this analysis as a predefined field. The contraction of volume occurs when ferrite was converted into austenite, while the expansion of volume occurs when the supercooled austenite was converted into ferrite during cooling caused by SSPT were also considered, and the evolution of compressive stress was preserved. Finally, the evolutions of temperature and compressive stress were input into the simulation of mass diffusion as predefined fields to obtain the hydrogen transient distribution in welding. The finite element commercial software ABAQUS-2016 (Dassault Systèmes Simulia Corp, Johnston, RI, USA) and subroutines (Uexpan and Ufield) were used in all the solution processes. The subroutines were written for the process of microstructure evolution. They were used to solve the process of microstructure evolution, temperature field, stress field and hydrogen distribution field of welding in the calculation process of material parameters. However, in the process of multi-pass welding, single pass welding involves the process of microstructure evolution about heating and cooling, and the entire welding process of the subsequent welding bead will also cause the phase change of the previous welding beads, so the influence of microstructure evolution on the hydrogen diffusion of multi-pass welding must be considered.

**Figure 3:**
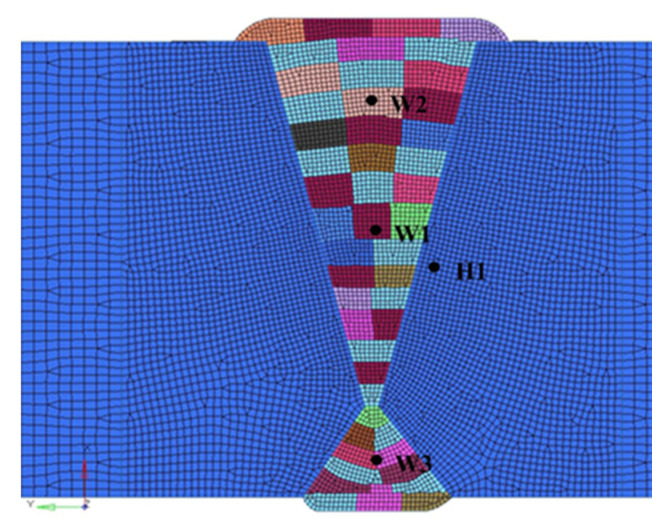
Welded joint grid model.

Specifically, according to the comparison of experimental results and results of simulation using a two-dimensional model in reference [20], the depth position where the amount of hydrogen concentration reaches its peak was different, the hydrogen distributions based on the results from the experiment and numerical analysis were not similar in shape slightly. ABAQUS was selected to simulate the coupling process of thermal-mechanical-hydrogen diffusion. The finite element model of the heavy plate welded joint was established as shown in Figure 3. The distribution of the welded joint was also shown in the figure. The finite element mesh was a four-node structure. Specifically, element types for thermal analysis and hydrogen diffusion include DCAX4, a 4-node linear axisymmetric heat transfer quadrilateral; stress analysis: CAX4R, a 4-node bilinear axisymmetric quadrilateral, reduced integration, hourglass control. The model establishment was consistent with the test results. The multi-pass welding was adopted, 36 passes were filled on the front side of the welded joint, and 13 passes were filled on the back side.

The chemical compositions in the weld filler was the same as that in base material, and it was assumed that the material properties of WM and BM were the same.

### 3.2. Phase Transformation

Following the concept of phase field which was one of microstructure simulation method [29,30,31], a user-defined field variable was used as a parameter to represent the phase state, and was invoked in subroutine of Ufield during the calculation. The user-defined field variable was defined as −1 for austenite state, while 1 for α-ferrite. According to the experimental results, the microstructure morphology and mechanical composition of multi-pass welded joints were relatively stable. The basic microstructures in HAZ and BM were ferrite and pearlite. From the continuous cooling transition (CCT) curve, the microstructure changes little with changing the cooling rate of welding, and the change range of thermal expansion was also small. Therefore, in the welding cooling process, the mixed microstructure of ferrite and bainite can be unified as α-ferrite. In the welding heating process, the temperature was higher than the actual temperature of phase transition (Ac3), the materials were in austenite state, and the user-defined field variable was changed from 1 to −1. In the welding cooling process, the undercooled austenite transferred to α-ferrite, the user-defined field variable parameter returned to 1 from −1. During the phase transformation of α-ferrite to austenite in the process of welding heating, the user-defined field variable was changed linearly with temperature, while the field parameter adopted a temperature dependent function during the transformation of undercooled austenite to α-ferrite in the process of welding cooling.

Based on the above definition, during the calculation of each physical field, the relevant material properties of the microstructure were called through the user-defined field variable. The material parameters of each phase are shown in Table 1 and Table 2 [32].

**Figure 4:**
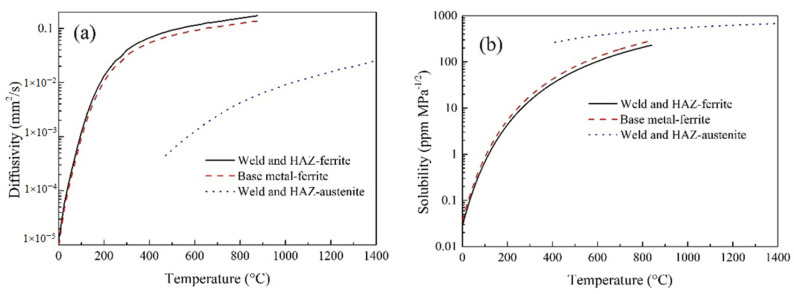
Parameters of hydrogen diffusivity in different phases: (**a**) Hydrogen diffusion coefficient; (**b**) Solubility [33].

The definition and invocation of hydrogen diffusion properties were also defined by the calculation results of the microstructure, that is, according to the phase state of the material at the current time and position. The property parameters of the hydrogen diffusion coefficient and solubility of different phases are shown in Figure 4 [33]. The characteristics defined in Figure 4 may not be precise enough, but they are sufficient to reveal the effect of SSPT on hydrogen diffusion in the weld.

**Figure 5:**
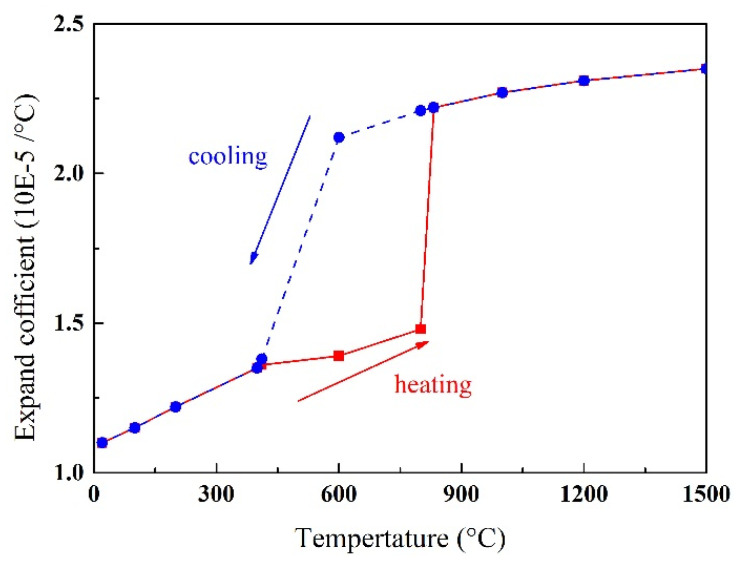
Description of thermal expansion coefficient.

**Figure 6:**
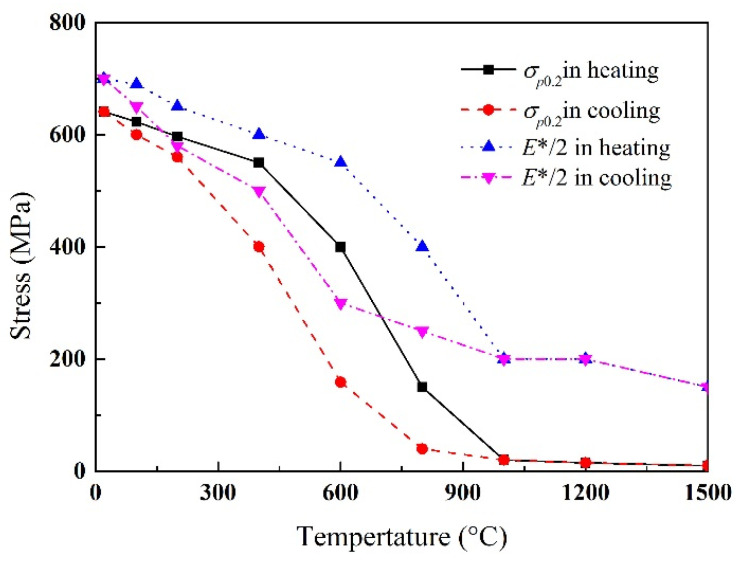
Yield strength and hardening modulus.

To directly present mechanical property change evolution in the welding process, the mechanical property parameters during heating and cooling processes are shown in Figure 5 and Figure 6.

According to the simulation process described above, the coupling model of thermal-mechanical-hydrogen diffusion was established. The phase transition points Ac1 and Ac3 are 795 °C and 900 °C during heating, respectively. According to the CCT figure [34] of the steel, martensitic transformation occurs in undercooled austenite during welding cooling. The phase transition starts and end temperature points, i.e., *Ms* and *Mf*, change with different cooling rates. In the cooling rate range studied in this work (the preheating process at 200 °C results in a limited range of *T*8/5 change in the welding cooling process), the *Ms* and *Mf* points have small fluctuations. For the convenience of research, set *Ms* = 430 °C and *Mf* = 280 °C. Martensitic transformation can be described by Koistinen–Marburger equation:

(1) $$\Delta F_{m}=F_{\mathrm{mod}}\left\{-k\exp\left[k\left(T-M_{S}\right)\right]\right\}\Delta T_{C}\cdot \left(M_{f}\leq T\leq M_{S}\right)$$

where $\Delta F_{m}$ is the transformation amount of martensite at different temperatures, *k* = 0.021 is the martensite constant, and $F_{\mathrm{mod}}$ is the constant, defined as:

(2) $$F_{\mathrm{mod}}=1/\left\{1-\exp\left[-k\left(M_{S}-M_{f}\right)\right]\right\}$$

The volumetric expansion strain was obtained by calculation:

(3) $$\Delta \varepsilon_{C}^{\Delta Vol}=\Delta F_{m}\varepsilon_{C}^{\Delta Vol}$$

where $\varepsilon_{C}^{\Delta Vol}$ represents the volumetric expansion strain when undercooled austenite transforms into martensite completely.

### 3.3. Simulation Procedure

#### 3.3.1. Thermal Analysis

In multipass welding, the precise prediction of phase transitions requires thermo-metallurgical analysis, including the process of calculating transient heat transfer and phase transition dynamics, which describe the variation of the phase region with temperature. According to Fourier’s law, the transient temperature field is calculated by:

(4) $$\frac{\partial }{\partial x}\left(\lambda \left(T\right)\frac{\partial T}{\partial x}\right)+\frac{\partial }{\partial y}\left(\lambda \left(T\right)\frac{\partial T}{\partial y}\right)+\frac{\partial }{\partial z}\left(\lambda \left(T\right)\frac{\partial T}{\partial z}\right)+q=\rho C_{P}\left(T\right)\frac{\partial T}{\partial t}$$

where *T* is the transient temperature field; ρ is density; *C_p_(T)* and *λ(T)* represents the temperature-dependent specific heat and thermal conductivity, respectively; *q* is the release intensity of the internal heat source due to SSPT, its changes with time and location are shown in Equation (5).

The relationship between the material and the temperature must be considered during the solution process. The flow field in the molten pool would accelerate the conduction of heat flow in the molten pool. In order to consider this phenomenon, when the material exceeds the melting temperature, set its thermal conductivity to a larger value. The volumetric heat source model used is as follows:

(5) $$q\left(x,y,z\right)=\frac{\eta UI}{V_{r}}\exp\left\{-3\left[\frac{v\left(\tau -t\right)}{Rv}\right]\right\}$$

where *q* represents the release intensity of heat source with time and location, *η* is the effective coefficient of the heat source. For SAW, 0.9 was generally adopted, *τ* is a delay coefficient used to define the central position of the heat source at time 0. *Vr* (mm^3^) is three-dimensional volume affected by heat source, the definition and the method of determining its value can be found in the literature [35,36]. *Rv* represents the influence parameter that influences the heat flow distribution.

In order to consider the heat dissipation during the welding process, the heat radiation and convection on the surface of the workpiece were considered during the solution. The boundary condition equation is:

(6) $$q_{c}=h_{f}\left(T-T_{0}\right)$$

(7) $$q_{r}=\varepsilon \sigma \left(T^{4}-T_{0}^{4}\right)$$

where *hf* is the coefficient of air heat transfer in W/$m^{2}$/°C, $T_{0}$ (°C) is the ambient temperature, *σ* is the coefficient of Stefan–Boltzmann, and *ε* is the coefficient of radiation.

#### 3.3.2. Mechanical Analysis

During the solution of stress and strain, some mechanical properties were defined as temperature-dependent. Among them, key mechanical properties such as thermal expansion coefficient and yield strength had a great impact on welding residual stress.

During the heating process, the material would change from ferrite to austenite in the temperature range of A1–A3. During the cooling process, the material would undergo supercooled austenite to ferrite transformation in the temperature range of Fs–Ff. The key mechanical property parameters should consider this solid phase transformation process. The thermal expansion coefficient of austenite was about twice that of ferrite. Its definition was divided into two processes, as shown in Figure 5, that is, the heating process and the cooling process were defined separately. The strength of austenite is significantly lower than that of ferrite. Similarly, the yield strength of the material was also positioned in two processes, as shown in Figure 6. The material constitutive model adopted the equivalent Mises yield criterion and the isotropic hardening rule. The hardening process was simplified to linear, yield strength and hardening modulus $\sigma_{p0.2}$ and E*, respectively.

**Figure 7:**
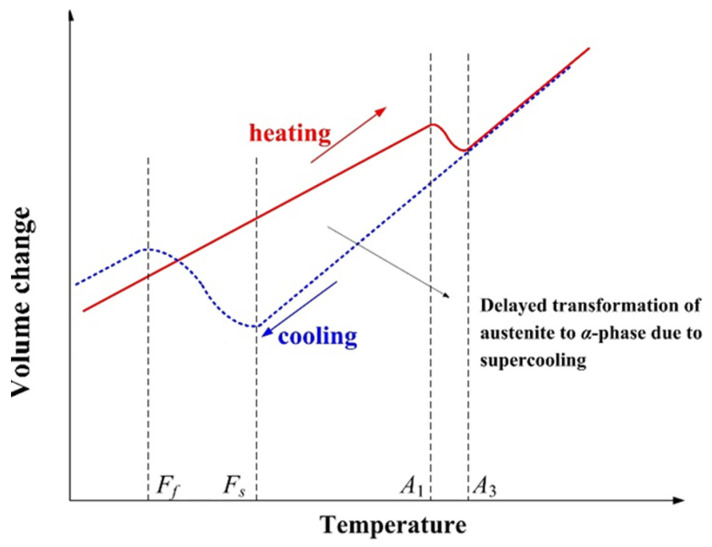
Schematic diagram of volume change during solid-state phase transition.

As shown in Figure 7, the solid phase transitions during welding heating and cooling processes cause volume constriction and expansion, respectively. The amount of expansion has a large impact on welding residual stress, which was also taken into account in the calculation process.

The volume constriction during heating is defined as:

(8) $$\Delta \varepsilon_{h}^{\Delta Vol}=\frac{\Delta T_{h}}{A_{3}-A_{1}}\Delta \varepsilon_{h}^{\Delta Vol*}$$

In the formula, $∆T_{h}$ represents the temperature increase of the heating process. The calculation results of A1 and A3 are 713 and 832 °C, respectively. $\varepsilon_{h}^{∆Vol*}$ is the volume expansion strain of all austenitization, and its value was defined as $-2.29\times 10^{-3}$. This shrinkage was only loaded under specific conditions, that is, when the temperature of the material was heated to between A1 and A3 during the heating process.

The volume expansion resulting from the decomposition of supercooled austenite during cooling is defined as:

(9) $$\Delta \varepsilon_{c}^{\Delta Vol}=\frac{\Delta T_{c}}{F_{s}-F_{f}}\Delta \varepsilon_{c}^{\Delta Vol*}$$

where $∆T_{c}$ is the temperature increase during the cooling process. *Fs* and *Ff* were obtained through experiments at 561 and 411 °C, respectively. $\varepsilon_{c}^{∆Vol*}$ is the volume expansion strain of austenite completely decomposed, the experimental measurement was $7.38\times 10^{-3}$. Similarly, the expansion amount was only loaded under specific conditions, i.e., the material was heated above A3 during the temperature rise process, and the cooling process was located within the temperature range of *Fs* and *Ff*.

#### 3.3.3. Hydrogen Diffusion Analysis

The driving force for hydrogen diffusion is the chemical potential gradient, that is:

(10) $$J=-\frac{Dc}{R(T-T_{z})}\frac{\partial \mu }{\partial x}$$

where *D* is the diffusion coefficient of hydrogen, *R* is the gas constant, *T* is the Kelvin temperature, *Tz* is the absolute zero degree, and *μ* is the chemical potential, which is defined as:

(11) $$\mu =\mu_{0}+R\left(T-T_{z}\right)\ln\phi +\sigma_{h}V_{H}$$

where $\mu_{0}$ is the chemical potential constant without considering the stress conditions, $V_{H}$ is the partial molar volume of hydrogen in the solid, and its value is $2{\;\mathrm{cm}}^{3}{\mathrm{mol}}^{-1}$ [37]. $\sigma_{H}$ represents hydrostatic pressure, *ϕ* = *c*/*s* represents hydrogen activity, and *T* is the temperature.

Taking no account of the temperature gradient, yields

(12) $$J=-sD\left[\nabla \varphi +k_{p}\nabla p\right]$$

where *k_p_* is the stress factor providing diffusion driven by hydrostatic pressure gradient, *k_p_* represents the compressive stress coefficient, and represents the stress gradient that drives the diffusion. It was defined as:

(13) $$k_{p}=\frac{V_{H}\phi }{R(T-T_{z})}mmN^{-1/2}$$

The mass conservation for diffusing phase requires

(14) $$\frac{dc}{dt}=-\nabla J$$

Inserting Equation (12) into Equation (14) yields

(15) $$\frac{d\varphi }{dt}=D\left[\nabla^{2}\varphi +\frac{VH}{RT}\nabla \varphi \nabla p+k_{p}\nabla^{2}{}_{p}\right]$$

Based on Equation (15), the transient diffusion of hydrogen is calculated by ABAQUS.

The stress-strain assisted diffusion equation predicts a higher amount of hydrogen in the critical area than the stress-only assisted diffusion or the conventional diffusion models [38], but in plastically strained material as welded joint of heavy plate, the decrease of hydrogen diffusivity predominates over the increase of hydrogen solubility due to accumulated plastic strain in highly strained materials. Hence, the strain is not considered in the hydrogen diffusion model of this research.

During the welding process, the ambient temperature of the molten pool is extremely high, and the hydrogen atom diffusion coefficient after ionization was extremely high. On the other hand, the molten pool flows quickly under the action of various physical field forces, and the hydrogen entering the molten pool is stirred to the entire molten pool. Therefore, it was assumed in the calculation process that when the molten pool was formed, a certain concentration of diffused hydrogen was uniformly distributed throughout the molten pool. In engineering, the hydrogen content of low-hydrogen welding materials was mostly distributed in the range of 1–4 mL/100 g, which was 1.12–3.57 ppm, according to the relevant hydrogen measurement standards. Considering the hydrogen diffusion during the cooling of the molten pool, it was assumed that the initial hydrogen content of the molten pool was 5 ppm, and the hydrogen content in the surrounding environment was defined as 0.

In order to consider the influence of the microstructure difference of the welded joints on the hydrogen diffusion, the positions of BM, WM and HAZ were judged by the temperature field calculation results, and then the hydrogen diffusion properties of the materials were given to each position.

## 4. Results

### 4.1. Simulation Results of Multi-Pass Welding of Heavy Plate

#### 4.1.1. Welding Temperature Field

**Figure 8:**
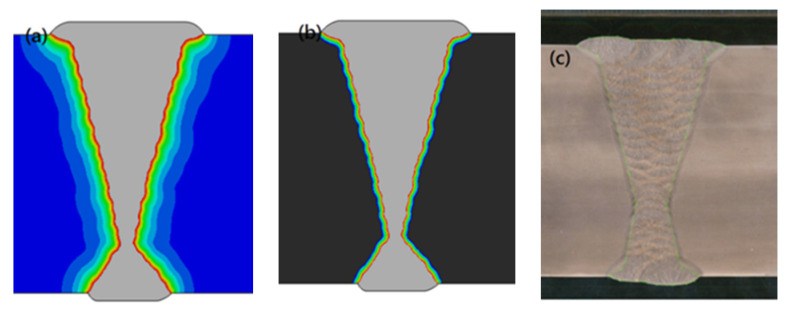
Comparison of Simulation Results and Experimental Results of Welded Joint of Heavy Plate: (**a**) Weld pool shape; (**b**) Morphology of HAZ; (**c**) Experimental results [20].

The results of the welding temperature field of the heavy plate are shown in Figure 8. The shape of the molten pool, the location and size of the HAZ in the welded joint were in good agreement with the experimental results, as shown in the Figure 8a, the part of gray was the melted part in the melting pool, which was beyond the melting point. Extending outwards, the red green and light blue areas showed cloud diagram in the heat affected zone, which was solid below the melting point. Figure 8b is the morphology of the welding heat affected zone. The black part was the position where the phase transition did not occur, and here, the temperature was below Ac1.The parts of color and gray were the position where the phase transition occurred, and the part of gray was the melting state where the temperature was above the melting point. By comparing Figure 8a,b, it can be seen that the welding joint area begins to reach the temperature of phase transition near the heat-affected zone and the phase transition begins at Ac1.

#### 4.1.2. Residual Stress Distribution

**Figure 9:**
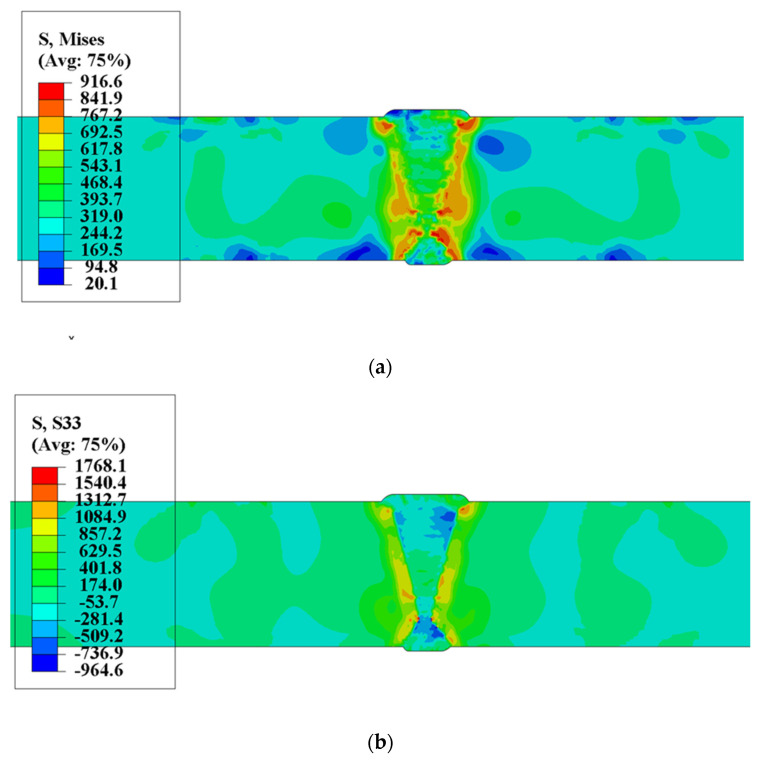
The result of residual stress of welded joint of heavy plate: (**a**) Equivalent Mises stress; (**b**) Longitudinal stress, (MPa).

The residual stress results of multi-pass welded joint of heavy plate are shown in Figure 9. The effect of volumetric expansion by SSPT of welded pass significantly changes the stress state of weld. As shown in Figure 9a the simulation results of the Mises stress, the stress concentration level of the capping bead was the lowest on both sides of the heavy plate. As the depth of the positive and negative sides increases, the equivalent stress concentration becomes higher, especially at the junction of the positive and negative welds. The stress level of WM, HAZ, and the BM near the HAZ was the highest. From Figure 9b, the results of longitudinal stress, it can be seen that most regions of WM were in the state of compressive stress. Due to the accumulation effect of multi-pass welding, the maximum compressive stress of positive and negative welds was located at a certain distance from the upper and lower surfaces, respectively. With the increase of the depth of both sides, the compressive stress level decreases, and the longitudinal stress of WM generates tensile stress at the junction of positive and negative welds. The HAZ of the welded joint was in the state of tensile stress, and the stress concentration in the thickness direction was uneven. The longitudinal tensile stress level was the highest at the position of weld toe and the junction of the positive and negative welds.

**Figure 10:**
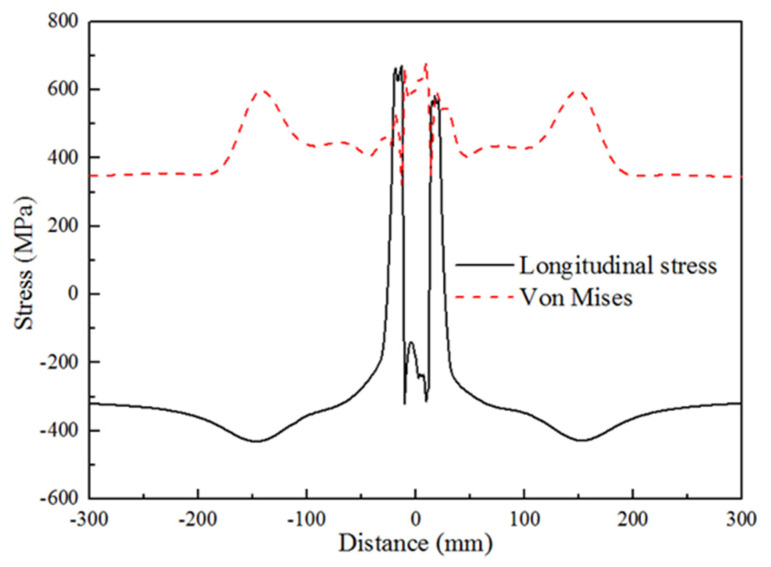
Residual stress-distance curve of heavy plate welded joint.

Figure 10 shows the distribution curve of the stress in the surface of the positive side along the transverse direction. It was obvious that the longitudinal stress was the compressive stress in WM, the equivalent stress and the longitudinal stress at the weld toe were the most serious stress concentration positions.

#### 4.1.3. Hydrogen Diffusion Behavior

**Figure 11:**
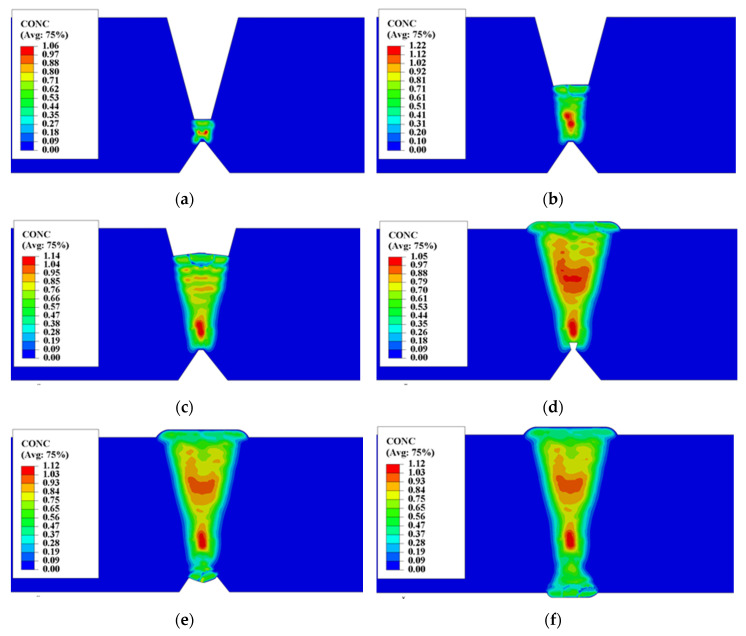
Distribution characteristics of hydrogen concentration (CONC) during welding of heavy plate: (**a**) 3rd weld pass front; (**b**) 11th weld pass front; (**c**) 23rd weld pass front; (**d**) Cosmetic bead front; (**e**) 6th weld pass back; (**f**) Cosmetic bead back.

The distribution of the hydrogen concentration during welding is shown in Figure 11. Most of the introduced hydrogen was distributed in the weld. Even though the preheating temperature and inter-pass temperature were high in the welding process of heavy plate, and the multi-pass welding process has a long period. However, the diffusion amount of hydrogen in the HAZ and the BM at this stage was very small. The hydrogen content of each weld pass was basically the same, but with the increase of the number of weld passes, the hydrogen was unevenly distributed along the thickness of the weld.

After the welding of the cosmetic bead, although the total introduced amount of hydrogen of the cosmetic bead was large, but the cosmetic bead surfaces directly contacted with the outside environment, and the contact area was large, so the diffusion amount of hydrogen into the outside was large, then cause the hydrogen concentration of the cosmetic layer was lower than that of the inner weld passes in a short time, as shown in Figure 11d,f.

After welding, there were two types of position with a high hydrogen concentration along the thickness direction, one was the position of weld heel, the other was the position of weld pass at a certain depth from the surface. Because the inter-pass temperature was controlled during the welding, the stress-induced hydrogen diffusion was not obvious, so the hydrogen distribution characteristics of the welded joint were formed by the combination of the welding thermal process and the self-gathering effect caused by the SSPT of each weld pass.

**Figure 12:**
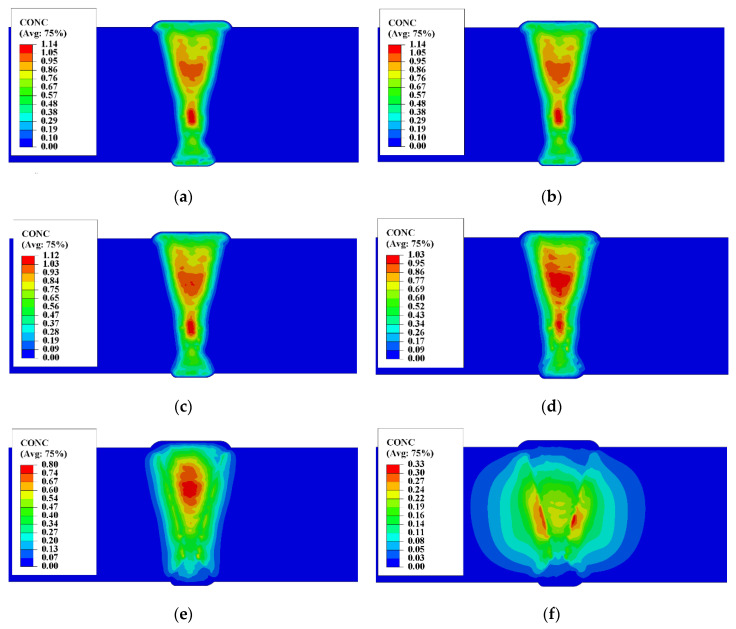
Distributing characteristics of hydrogen concentration (CONC) during cooling of heavy plate welding: (**a**) 1 h; (**b**) 10 h; (**c**) 100 h; (**d**) 1000 h; (**e**) 10,000 h; (**f**) 100,000 h.

The hydrogen distribution in the cooling process of welded joint is shown in Figure 12. Due to the large size of heavy plate and the number of weld pass, the total introduced amount of hydrogen the welded joint was large during the welding, so the escape of hydrogen to the outside needed a long-range diffusion, which took a long time. On the other hand, the welding residual stress was concentrated in the welded joint, the stress-induced diffusion hindered the free diffusion of hydrogen based on the difference of concentration of hydrogen, so the hydrogen concentration and diffusion trend at the aggregation position did not change much over a considerable period of time. It can be seen from the calculation results that the peak value of hydrogen concentration in the welded joint drops to 1 ppm when it was cooled to 10,000 h.

**Figure 13:**
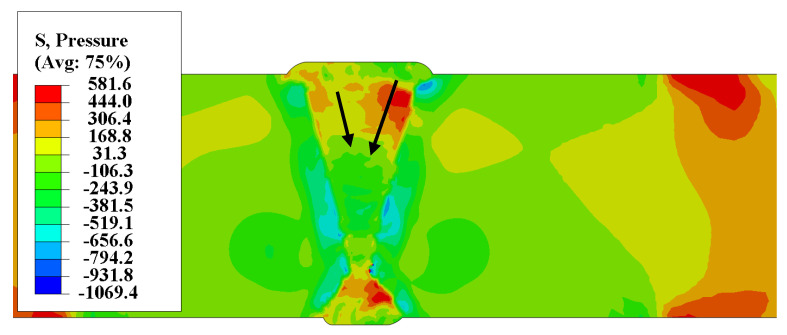
Compressive stress distribution of welded joint of heavy plate.

The stress induction had significant effect after the welded joint cools down. As shown in Figure 13, the results of the compressive stress show that the penultimate and third layers of the weld pass show a positive value on the front of the welded joint, that was the compressive stress state. When the depth increases, it shows a tensile stress state. Therefore, hydrogen diffused deeper from the direction below the weld bead, as shown in Figure 13 for black arrows. Therefore, the peak concentration of hydrogen in the middle position of the front weld fell very slow, and even increase occurred, as shown in Figure 12b–e.

The HAZ of the whole welded joint was the most severe position of stress concentration. The mechanism of residual stress induced hydrogen diffusion of heavy plate was consistent with that of multi-pass welded joint. The hydrogen first accumulated at the position of HAZ between welded passes in WM, and then accumulated to the HAZ of the whole welded joint. However, due to the size effect of heavy plate and the distribution of residual hydrogen, the required time (>50,000 h) for the hydrogen aggregation of HAZ in 100 mm heavy plate was much greater than that (>100 h) in 20 mm plate [33]. In addition, under the natural cooling condition, the residual hydrogen in the heavy plate joint diffuses to the outside, which caused the time for concentrations in the aggregation area to drop to low levels took years. It was much longer than the dehydrogenation time (several days) of the medium or thick plate. So, the dehydrogenation must be carried out during the welding of the heavy plate in engineering.

## 5. Discussion

### 5.1. Comparison of Results

**Figure 14:**
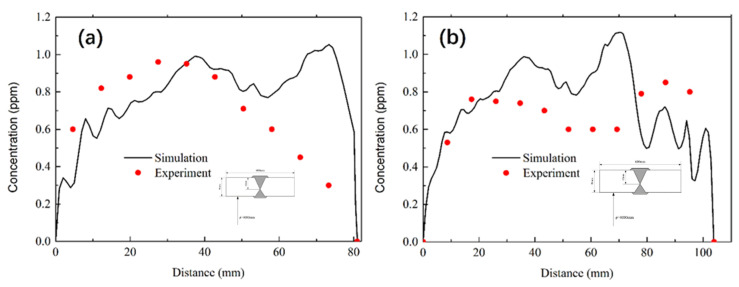
Comparison between simulation and test results of hydrogen concentration in thickness direction of heavy plate: (**a**) End of front welding; (**b**) End of back welding.

The comparison between the simulation results and the test results [20] is shown in Figure 14. The hydrogen concentration distribution in the thickness direction was in good agreement with the test results. However, there was little deviation in the local position. At the junction of the front and back welds, the simulation results showed that the hydrogen concentration in this position was high, but there was no hydrogen accumulation at this position in the test results. The reasons for the deviation may be as follows: (1) The test result was the average value of a bulk, while the value range of simulation was small. (2) In actual welding, back gouging was required for reverse welding, carbon arc gouging took off a considerable amount of materials of heavy plate, so as to take away part of hydrogen, but the simulation process did not consider this operation. (3) The simulation process was an ideal state, but the welding time, cooling time, and low temperature environment during hydrogen measurement would influence the test results in the actual welding process. (4) So greater were the hydrogen diffusion factors, the calculation model could not fully reflect the actual situation, such as high temperature material parameters, the complexity of the solid-state phase transformation (this paper only considers the transformation about austenite to ferrite), the initial concentration of hydrogen, and so on. Therefore, there was a certain error between the simulation results and the experimental results. In the thickness direction, experimental results show that the position with the highest hydrogen concentration was located at a position with 30–40 mm from the upper surface, which was consistent with the simulation results. Moreover, literature shows that cracks were initiated at a distance of 9.5–10.0 mm away from the top of the welded surface in the specimen welded at full thickness. [39].

### 5.2. Self-Gathering Effect of Welding of Heavy Plate

According to the research [33], there were two main aspects of the influence of SSPT on hydrogen diffusion. One was that SSPT reduces the residual stress of WM significantly, while increasing the residual stress of HAZ, and redistribution of hydrogen under the influence of stress [40]. The other was that in the process of SSPT, material properties of hydrogen diffusion change dramatically during a short time, the hydrogen diffusion coefficient increases by several orders of magnitude and the solubility decreases by several orders of magnitude. It directly led to the uphill diffusion of hydrogen in WM, which was the self-gathering effect.

The self-gathering effect of hydrogen in the weld pass caused the redistribution of hydrogen in the weld pass, and this effect between the weld passes made hydrogen diffuse from the last weld pass to the current weld pass. The results showed that the residual hydrogen of the subsequent weld pass after welding increases continuously due to the self-gathering effect between the weld passes in the multi-pass welding. However, in the welding of heavy plate, if the number of weld pass is large, it is worth exploring whether this mechanism holds.

**Figure 15:**
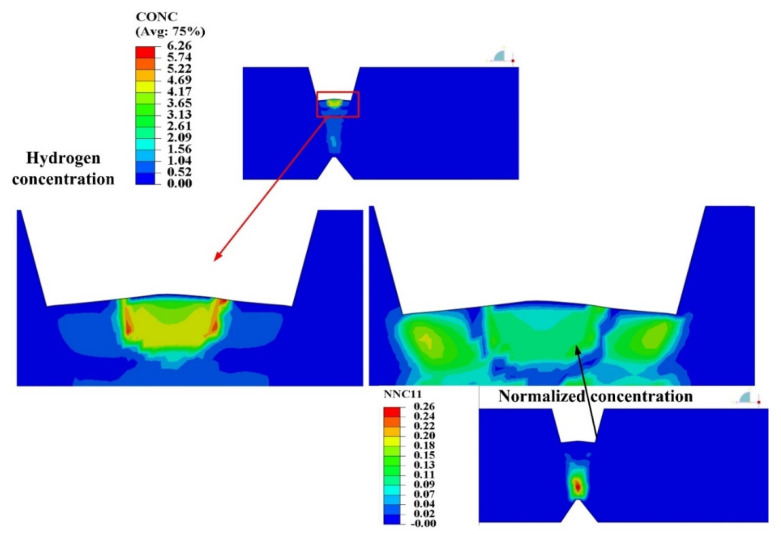
Self-gathering effect of welding process of heavy plate.

As increasing the weld pass, each weld pass of heavy plate has self-gathering effect. As shown in Figure 15, when the SSPT occurred in the assembly of weld pass, the hydrogen concentration in the internal local position exceeded the initial concentration of the weld molten pool. During the heating and cooling process of the weld pass, before the austenite phase was decomposed, the hydrogen of the previous pass and the previous layer diffused into the current weld pass continuously, forming the hydrogen accumulation at the junction of the weld pass with the previous weld pass. As can be seen from the activity distribution of normalized concentration (NNC11) in Figure 15, the activity values of the previous weld pass and the previous weld layer of the current weld pass were greater than that of the current weld pass, but the position of maximum activity value was at a certain distance away from the current weld pass, that was the position of the weld pass with a small serial number. Because of the low temperature at this location, the current weld pass has a small influence on its welding heat, and the material remains in the ferrite state and with a small solubility of hydrogen.

**Figure 16:**
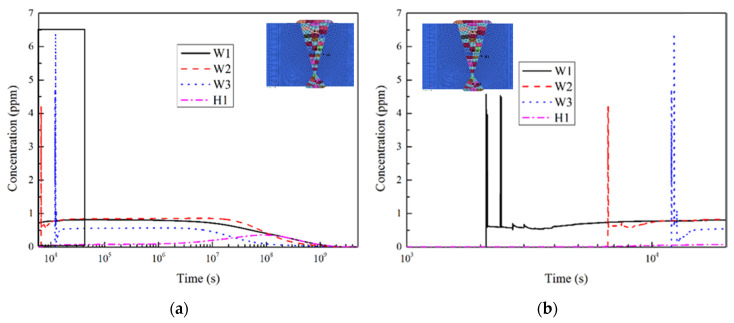
Concentration-time distribution curve of hydrogen of heavy plate welded joint: (**a**) Overall drawing; (**b**) Partial drawing.

The hydrogen concentration evolutions at some local points of WM and HAZ, as marked in Figure 1b, is shown in Figure 16, it can be seen that at different positions in the WM of welded joint, in addition to the high hydrogen concentration in the molten pool, there were uphill diffusions caused by the self-gathering effect during the cooling process or affected by the subsequent weld pass. And two effects were different. As shown in Figure 16b, material at position *W*3 experienced a more obvious self-gathering effect, while the effects of the self-gathering effect at the two points *W*1 and *W*2 were smaller.

### 5.3. The Effect of Self-Gathering Effect between Passes in the Thickness of Heavy Plate

**Figure 17:**
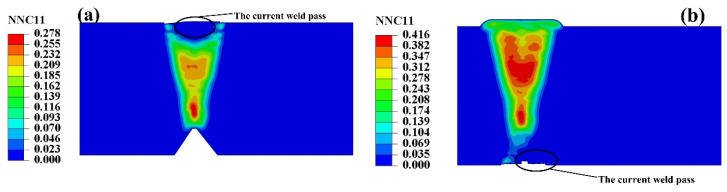
Activity distribution in welding process of heavy plate: (**a**) Front weld; (**b**) Back weld.

Theoretically, in the multi-pass welding of heavy plate, on the one hand, the source of hydrogen of self-gathering effect in the welding process of subsequent weld pass includes not only the previous weld pass, but also the weld pass of the previous layer; on the other hand, during the temperature decrease, the activity of residual hydrogen significantly increases with time in the previous one weld layer or more weld layers, which result in increasing activity gradients and then conducive to the diffusion of hydrogen between the weld passes. Then, from the calculation results, the effect of self-gathering effect between passes was limited. As shown in Figure 11 and Figure 13, it is obvious that the residual hydrogen concentration in the capping layer and its several nearby inner weld layers was neither in the enrichment region nor the peak concentration, no matter on the front or the back side of the heavy plate. This phenomenon is opposed to the effect of inter-pass self-gathering, which means the self-gathering effect between weld passes was not continuously effective in the direction of the thickness of the heavy plate. The reason is as shown in Figure 17, when welding in front weld or back weld, the activity of the weld pass with a larger depth distance from surface was greater, even more than an order of magnitude greater than the current activity around the current weld pass. However, due to the large distance, the temperature of the material with high activity always stays in a lower range, and the diffusion coefficient of hydrogen was small. Long-range diffusion cannot be achieved even under conditions of large activity gradients, which is consistent with the fact that it took much longer time for hydrogen to diffuse out of the welded joint for heavy plate than for middle and thin plate. However, within the range of adjacent weld passes, the current weld pass can still obtain the source of hydrogen diffusion from other previous weld passes.

Therefore, for the weld joint of heavy plate, the self-gathering effect between weld passes was effective in the short-range and ineffective in the long-range.

## 6. Conclusions

The thermal-mechanical hydrogen diffusion coupling model was established for the multi-pass welding process of heavy plate. The hydrogen diffusion behavior in the whole welding process was simulated. The results of hydrogen distribution were compared with the experimental results. The conclusions are as follows:

(1) The simulation results of distribution trend of hydrogen concentration along the thickness direction of the welded joint of heavy plate were in good agreement with the experimental results, and there were certain deviations in the results of local position.
(2) The size effect and large number of welded passes of heavy plate results in the total introduced amount of hydrogen from the outside being large. So, the time required for natural dehydrogenation after heavy plate welding was much longer than that of middle and thin plates.
(3) The simulation results and experimental results show that the position of the weld pass with a 20–30 mm distance from the surface in WM has the largest hydrogen content, and it was maintained at a high level for a long time. Thus, the position has the largest sensitivity of cold cracking. This phenomenon is consistent with existing research results.
(4) During the welding of heavy plate, the self-gathering effect between the weld passes was effective in the short-range and in the long-range, invalid.

## Figures and Tables

**Table 1:** Material properties of α—ferrite.

Material Properties	Temperature (°C)						
	20	100	200	400	600	700	850
Conductivity (W/m°C)	54.4	54.0	52.8	47.7	40.0	33.4	27.3
Specific heat (J/kg°C)	423	473	478	492	530	650	682
Young’s modulus (GPa)	215	207	204	185	166	134	118
Harden modulus (GPa)	1.4	1.3	1.3	1.2	1.1	1.1	1.0
Yield stress (MPa)	641	623	597	550	451	275	98
Thermal expansion coefficient (1 × 10^−5^)	1.10	1.15	1.22	1.37	1.42	1.46	1.49
Density (g/cm^3^)	7.81	7.79	7.77	7.72	7.69	7.66	7.61
Possion’s ratio	0.29	0.30	0.30	0.30	0.31	0.32	0.32

**Table 2:** Material properties of austenite/undercooled austenite.

Material Properties	Temperature (°C)						
	400	600	700	800	900	1200	1400
Conductivity (W/m°C)	18.0	19.2	22.5	24.0	28.1	32.2	34.0
Specific heat (J/kg°C)	540	570	584	608	632	676	700
Young’s modulus (GPa)	167	163	156	145	132	60	10
Harden modulus (GPa)	1.4	1.3	1.1	0.9	0.8	0.5	0.3
Yield stress (MPa)	160	141	123	101	60	25	10
Thermal expansion coefficient (1 × 10^−5^)	1.8	1.9	2.0	2.0	2.0	2.1	2.2
Density (g/cm^3^)	7.75	7.66	7.61	7.56	7.46	7.37	7.32
Possion’s ratio	0.32	0.32	0.33	0.33	0.33	0.34	0.39
